# A comparative study of antibacterial and antifungal activities of extracts from four indigenous plants

**DOI:** 10.6026/97320630016267

**Published:** 2020-03-31

**Authors:** Rajendra Mehta, Suraj Dhruv, Vidyanshu Kaushik, Kamal Kumar Sen, Naureen Shaba Khan, Amar Abhishek, Ashwini Kumar Dixit, Vibhay Nath Tripathi

**Affiliations:** 1 Department of Rural Technology, Guru Ghasidas University, Bilaspur, India 495009; 2 Department of Botany, Dr. C. V. Raman University, Bilaspur, India 495113; 3 Department of Botany, Guru Ghasidas University, Bilaspur, India 495009

**Keywords:** Antibiotic sensitivity, Mentha spp, Aloe vera, Nerium oleander, Eucalyptus spp

## Abstract

A repetitive and wide use of chemical antibiotics has brought a serious threat in the biomedical and clinical sectors by the emergence of multidrug resistant pathogens. Plants have secondary metabolites that make them suitable candidate for natural antimicrobial agent without any side effect. In this study, we assessed comparative antibacterial and antifungal effects of extracts from four Indigenous plants (Nerium sp; Mentha sp; Aloe vera and Eucalyptus sp). Total phenolic and flavonoid content were extracted by microwave-assisted extractor and used for phytochemical assay. Antimicrobial experiment was done by micro dilution technique. A post hoc analysis inbuilt with one-way ANOVA test was used for the compilation of antibiotic sensitivity data and percent inhibition. Total phenolic content was significantly high in Mentha sp. and low in Nerium sp. (All p < 0.05). In antibacterial and antifungal activity higher concentrations of extracts showed a strong activity, which was as good as antibiotics used as control. Results from Eucalyptus sample showed a significant growth reducing capability even at lower concentrations. This study concludes that the plant extracts can be used to treat microbial infections with almost same efficacy as antibiotics and with a lower chance of resistance development.

## Background

Microbial diseases, especially of bacterial origin are very prevalent. A large number of people get sick due to microbial infections every year. This causes a big burden on any country's social as well as financial health. Empiric mode of treatment, which use a repetitive and wide use of antibiotics, has brought a serious threat in the biomedical and clinical sectors by the emergence of multidrug resistant pathogens. Such cases has given an open opportunity to organisms and compelled them to synthesize different types of β-lactamases due to which the efficiency of β-lactamases has been challenged and led to parlous outbreak of drug resistant organisms causing tremendous harm to the human beings [1-4]. The administration of antibiotics in the viral infections hampers the immune system of the body and sometimes offers the normal flora of human body to become opportunistic and cause chronic problems [5,6].The plant extracts have immense potential to combat bacterial, fungal, protozoan and viral diseases without any known side effects. Presence of secondary metabolites such as flavonoids, alkaloids, tannins, and terpenoids make them suitable candidate among antimicrobial agent [7]. They are shown to exhibit antimicrobial properties against a wide range of Gram-positive as well as Gram-negative bacteria [8,9]. Elizabeth et. al, [4], studied the antibacterial activity and phytochemical screening of Dialium guineense seed extract against enteric bacteria. Yaraksa, [3] reported the use of five different medicinal herbs (i.e. Tristaniopsis burmanica, Capparis zeylanicaLinn., Markhamia stipulata, Caryota maxima and Amphineurion marginatum). Their applicability has not only confined to the antimicrobial properties, rather they also possess anti-inflammatory activity.

Different types of chemicals have been used to extract the active ingredients from different plant parts. Alam et al [10] investigated the antibacterial activity of methanol extract as well as the extracts obtained by using petroleum ether, chloroform and ethyl acetatefrom the root bark of Akanda (Calotropis gigantea). They showed that it had significant anti-bacterial activity against most of the bacteria tested. Similarly, antifungal activities of saponins from Tribulusterrestris L. were tested against Candida albicans, C. glabrata, C. parapsilosis, C. tropicalis and Cryptococcus neoformans. Results showed that saponins had significant antifungal activity and kill fungi by destroying the cell membrane [11]. The evaluation of antibacterial activity of Menthaspicata and Menthapiperita essential oils were found to effectively inhibit the clinical isolates tested [12]. Many essential oils including those from Eucalyptus have been used in folk medicine throughout the world, and their medicinal properties have been investigated. Essential oils from Eucalyptus sp. exhibit antibacterial, antifungal, analgesic and anti-inflammatory properties and have been widely used in pharmaceutical, food, and cosmetics products [13]. Although different researchers had reported significant uses of plant extracts against different microbes, a lot is still left unexplored. Keeping in view the aforementioned facts present study has been conducted to see the effect of methanolic extract of leaves of four medicinal plants (i.e. Mentha spp., Aloe berbadensis, Nerium oleander and Eucalyptus spp.) against a pathogenic bacterial and fungal species i.e.(S. aureus and C. albicans) respectively.

## Materials and Methods:

### Collection of plant and extraction of secondary metabolites: 

Mentha spp, Ecalyptus spp, Aloe barbadensis and Nerium oleander were collected from natural habitat of Guru GhasidashVishwavidyalaya campus Bilaspur Chhattisgarh, India.The selected plant parts were used for extractions of phenolic and flavonoid content with microwave assisted extractor (MAE) and used for phytochemical assays. MAE combines microwave and traditional solvent extraction, which increases the kinetic of extraction. MAE has a number of advantagesover traditional methods of extraction, e.g., shorter extraction time, less solvent requirement, higher extraction rate and lower cost [14].

### Phytochemical Assay:

Total Phenolic contents (TPC) and Total Flavonoid Content (TFC): Total phenolic content was determined by the foiln ciocalteau assay [15] as described below. 1ml of oil extract was mixed with 5ml of 10% folinciocalten reagent and 4ml of sodium carbonate (7.5%) solution. The mixture was inoculated for 20 min at room temperature followed by absorbance measurement at 760 nm. Gallic acid solution was used for preparation of standard curve and the phenolic content of sample was expressed as mg of GAE (Gallic acid equivalent) /gm of the extract.

Total flavonoid content was measured by the aluminium chloride colorimetric assay [16]. 1 ml off oil was diluted with 4ml of deionised water. Sodium nitrate (300 µl of 5% solution) was added for 5 minutes followed by addition of aluminium chloride (300 µl of 10% solution) for 6 minutes. 2 ml of 1M NaOH was added and final volume was made up to 10 ml with distilled water. The solution was mixed well and absorbance was measured at 510 nm. The total flavonoid content was expressed as mg quercetin equivalents.

### Antimicrobial Assay

#### Chemicals and microbial media:

Ciprofloxacin as well as nutrient growth medium (Nutrient agar, nutrient broth, yeast peptone broth etc.) were obtained from Hi-media. Nutrient agar and nutrient broth media were used to test the growth of bacteria, while yeast peptone broth was used for growth of fungal species.

#### Microbial strains:

Staphylococcus aureus (MTCC 96) and Candida albicans (MTCC 227) were used for the antimicrobial studies and minimum inhibitory concentration (MIC) determination. Preparation of bacterial inoculums was done as per Wiegand et al.[17], while fungal inoculums prepared according to Al-Hatmi et al.[18].

#### Antimicrobial activity and determination of MIC:

Oils extracted from different plant leaves containing phenolics and flavonoids are used in the study to determine the antimicrobial activity and to calculate minimum inhibitory concentration. A 50 µl test isolates were pipette grown in 96 well ELISA plate (EP1-5x10no, Himedia). Final extract of each plant was considered as their stock (D1: (µg/well). The D1 of Nerium, Mentha, Aloe vera and Eucalyptus are 58, 55, 59 and 58, respectively. Further two dilutions D2 (half of D1) and D3 (half of D2) were prepared by dilution of their respective stock. These three different dilutions (D1 to D3) of the oil samples were used for determination of MIC. The experimental plates were incubated at 35°C for 24 h and 25°C for 72h for bacteria and fungi, respectively. The observations were recorded after 24h and 48h in case of bacteria in an ELISA plate reader. Whereas, fungal growth was recorded after 72 hours of incubation period. There are two controls were used, First growth control and second positive control (antibiotics: 50µg/ml). Positive control was used to compare the effectiveness of drug and growth control was used to observe the effect of the process of antibiotic activity.

### Statistical analysis:

For continues data, normality was tested using Kolmogorov Smirov test. Numerical data were presented in the form of mean and SD. A post hoc analysis inbuilt with one-way ANOVA test was used for the compilation of antibiotic sensitivity data and percent inhibition. The level of significance that means, p value was set as <0.05. The help of SPSS prepared graphs.

## Results and discussion:

In this study, four local plant species were used for a comparative analysis of their antimicrobial efficacy. These plants belong to different families and found locally in the area. Local ethnic people to treat infections use crude extracts from these plants.

## Total Phenolic and Flavonoid content:

**Figure 1:**
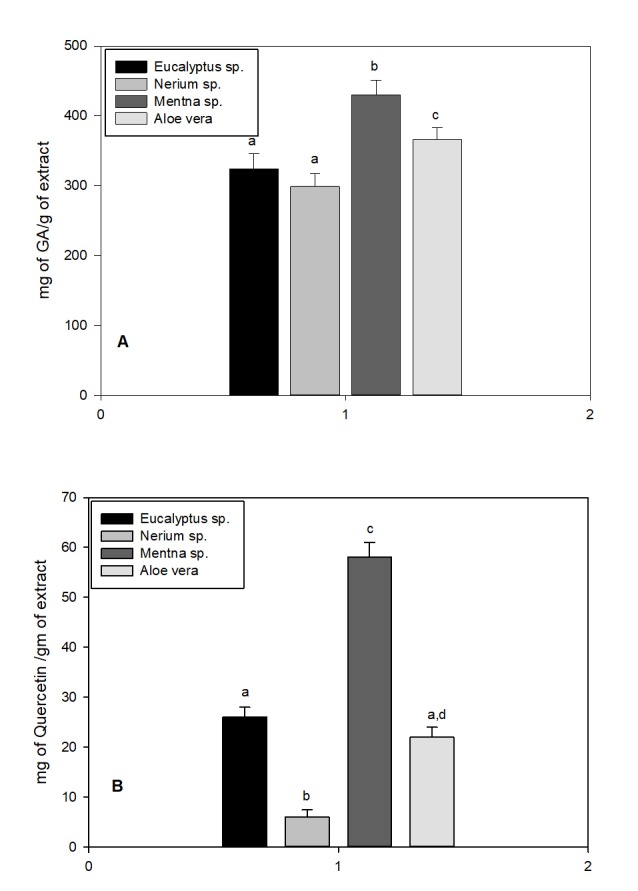
Total Phenolic (A) and Total flavonoid content (B) of four different plants. Different small alphabets in separate section (A or B) showed significant variation. Significance was calculated by post hoc LSD one way ANOVA at p < 0.05.

Phenolics and flavonoids are two important phytochemicals reported from different medicinal plants. These compounds mainly help the plant in their response to many stress conditions. The total phenolic contents of the extracted essential oils were examined using the Folin-Ciocalteu method and expressed in terms of gallic acid equivalent. The results of the finding are illustrated in (Figure 1A).Total phenolic content was significantly highest in Mentha sp. and lowest in Nerium sp (All p < 0.05). The concentrations of flavonoids in essential oilextracts were determined using spectrophotometric method with Aluminium chloride (AlCl3) and expressed in terms of Quercetin equivalent (mg of Quercetin /gm of extract). The concentration of flavonoids in different plant extracts are shown in (Figure 1B).Concentration of flavonoids is low when compared to total phenolic contents. It was as expected, as flavonoids are generally present in very low concentrations in plants. Concentration trend of flavonoids in four experimental plants are similar to concentration trend for totals phenolics. It was highest in Mentha sp. and lowest in Nerium sp (All p < 0.05). Concentration of flavonoids in Eucalyptus sp. and Aloe vera is similar (p > 0.05) and they are in between Mentha and Nerium sp (p < 0.05).

## Antimicrobial activity:

**Figure 2:**
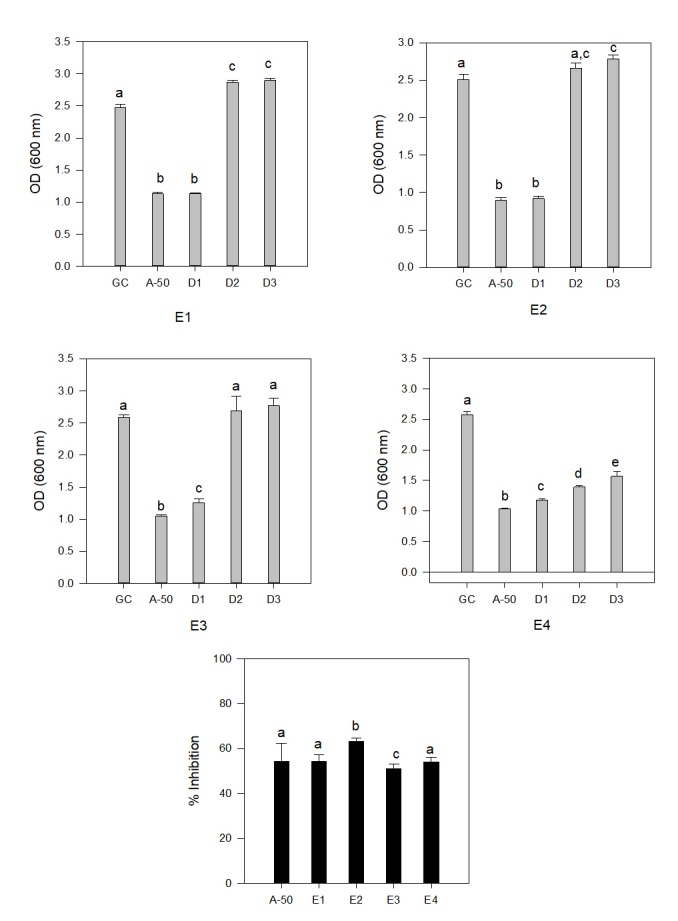
Distribution of anti bacterial effect of different plant extract (E1: Nerium sp; E2: Mentha sp; E3: Aloe-vera; E4: Euclyptus) and their % inhibition (GC: growth control; A-50: Ciprofloxacin 50 µg/ml). Different alphabets in separate plants (E1-E4) showed significant variation. Significance was calculated by post hoc Tukey one way ANOVA at p < 0.05.

**Figure 3:**
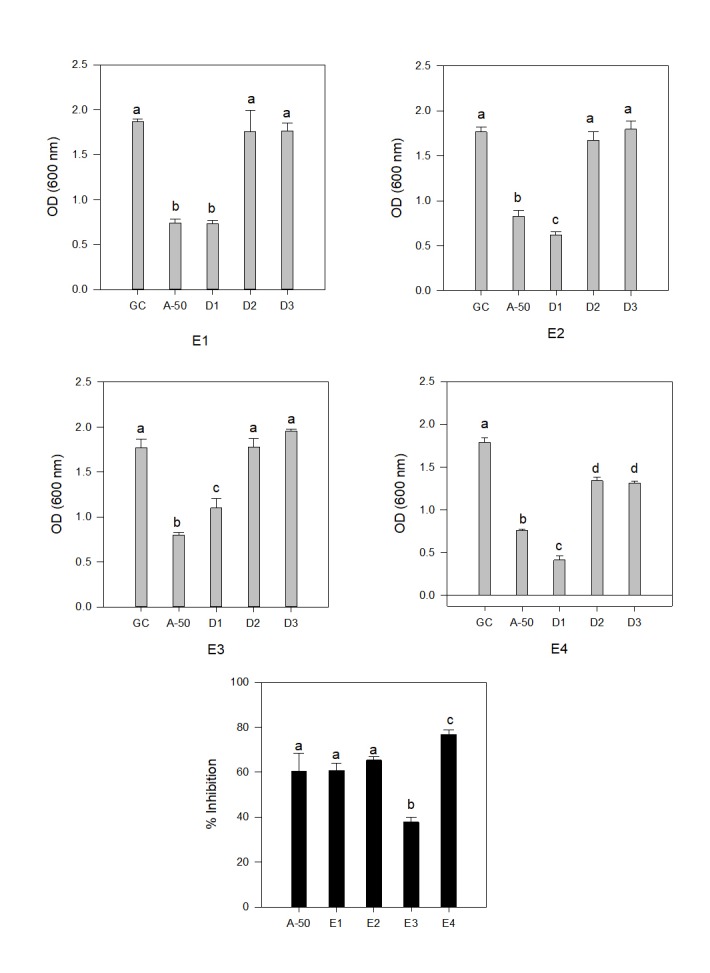
Distribution of anti fungal effect of different plant extract (E1: Nerium sp; E2: Mentha; E3: Aloe-vera; E4: Euclyptus) and their % inhibition (GC: growth control; A-50: Ciprofloxacin 50 µg/ml). Different alphabets in separate plants (E1-E4) showed significant variation. Significance was calculated by post hoc Tukey one way ANOVA at p < 0.05.

Essential oils containing phenolics and flavonoids were extracted from Nerium sp. (E1), Mentha asp. (E2), Aloevera (E3) and Eucalyptus sp. (E4) and were assessed for their antimicrobial activities against bacterial and fungal species. The experiment was done in 96 well plate by micro dilution technique. Proper controls were taken in different wells. After incubation, absorbance was taken and average results were presented in (Figure 2 and Figure 3). In (Figure 2), the result obtained from treatment against a bacterium is shown. Bacterial strain chosen for this study was Staphylococcus aureus, as it is a commensal bacterium and also a major human bacterial pathogen with wide variety of clinical manifestation [19]. S. aureusis Gram positive bacteria with coccus shape. It is sensitive to antibiotic, which was used as positive control in this experiment [20,21]. In case of Nerium sp. (E1), higher concentrations of plant extracts showed a strong activity against the pathogen. At higher concentrations, activity was as good as antibiotics used as 50µg/ml. At lower concentrations, the activity against the pathogen is negligible and growth is not hampered (Figure 2). A similar result was seen in case of Mentha Sp. (E2) and Aloe vera (E3) extracts. In all the three cases higher concentration was working efficiently to kill the bacterial pathogen, but the lower concentrations were not as efficient. Sometimes, it even looks like that lower concentrations are favouring the growth of the organism (Figure 2). This may be attributed to the chemistry of the active ingredients in essential oils, where biological properties i.e. (antimicrobial capability) get altered and instead of growth inhibiting, they transform as growth promoting stuffs. Results from essential oil extracted from Eucalyptus sample (E4) were different. It showed a significant growth reducing capability even at lower concentrations. In case of Eucalyptus oils, higher as well as lower concentrations were effective killer of the bacteria and showed an activity similar to antibiotics used as control. Thus, we can say that Eucalyptus essential oil has a great potential in decreasing the growth of gram-positive bacteria. Interestingly though, when all the four essential oils were compared at higher active concentrations, results did not show Eucalyptus as the best among four (Figure 2). In this comparison, Mentha sp. showed significantly greater growth retarding capability, which was better than even the antibiotics used as positive control. Similar experiment was performed to see the efficacy of these essential oils against a fungal pathogen, results of which is present in (Figure 3). Candida albicans was the ideal choice for this study, as this fungus residesas a harmless commensal with most of the humans. However, under favourable condition, C. albicans can cause infections ranging from superficial infections of the skin to life-threatening systemic infections [22]. Results presented in (Figure 3), showed similar antifungal activity as was shown for antibacterial activity. All the four plant extracts showed great response at higher concentrations, when compared to lower concentrations. Growth inhibition at higher concentration was comparable to, or even better than control antibiotics. Lower concentrations used for different essential oils were showing non-significant difference from growth control in three out of four cases. Only in the case of Eucalyptus oil, even the lower concentrations showed growth inhibition. Eucalyptus oil was the best antifungal agent among the four plant extract tested, even when we compared the higher concentrations of all four extracts. The activity shown by Eucalyptus oil against C. albicans was significantly better than the positive control (antibiotics). Different groups from different countries has done related work and showed similar promising results. Gulluce et al.[23] also performed the antimicrobial and anti-oxidant properties of essential oil from menthalongifolia and found that essential oil possesses strong antimicrobial properties against 30 isolates. Kiran and Prasad, [24] also performed the antimicrobial studies of Nerium oleander leaf by extracting them using cold maceration extraction method in methanol and chloroform employing well diffusion method against different bacterial and fungal isolates viz. E. Coli, B. subtilis, A. brasiliensis and C. albicans. Eucalyptus essential oil antimicrobial activity and found antibacterial activity with varying magnitudes, depending on the size of inoculums and the concentration of essential oil. Hassan et al.[25] studied the antidiarrhoeal; antimicrobial and cytotoxic activities of ethanol-extracted leaves of yellow oleander (Thevetiaperuviana) and found poor antimicrobial activity of the plant extract. Bachir and Benali,[26] examined the in-vitro antimicrobial activities of essential oil of the leaves of Eucalyptus globulus. The results obtained showed that essential oil of the leaves of E. globules has antimicrobial activity against gram-negative bacteria (E. coli) as well as gram-positive bacteria (S. aureus).

## Conclusions:

Results presented in this paper are in agreement with the earlier observations. In this study essential oils extracted from four different plants were used as crude drugs with high efficacy. Local ethnic people to cure different infections, from many generations, use all these plants. This study validates this ethnic knowledge, as the plants were collected from the same locality. Essential oils used in this study have a mixture of chemicals, meaning that developing resistance against them will be hard for the pathogens. If, in future, efficacy of the drug needs to be increased, active ingredients will be identified and used.
